# Host Antimicrobial Peptides in Bacterial Homeostasis and Pathogenesis of Disease

**DOI:** 10.3390/antibiotics3040645

**Published:** 2014-11-17

**Authors:** Derek R. Heimlich, Alistair Harrison, Kevin M. Mason

**Affiliations:** 1 The Research Institute at Nationwide Children’s Center for Microbial Pathogenesis, Columbus, OH 43205, USA; E-Mails: derek.heimlich@nationwidechildrens.org (D.R.H.); alistair.harrison@nationwidechildrens.org (A.H.); 2 The Ohio State University College of Medicine, Department of Pediatrics, Columbus, OH 43205, USA

**Keywords:** antimicrobial peptides, pathogenesis, bacteria, microenvironment, therapeutics

## Abstract

Innate immune responses function as a first line of host defense against the development of bacterial infection, and in some cases to preserve the sterility of privileged sites in the human host. Bacteria that enter these sites must counter host responses for colonization. From the host’s perspective, the innate immune system works expeditiously to minimize the bacterial threat before colonization and subsequent dysbiosis. The multifactorial nature of disease further challenges predictions of how each independent variable influences bacterial pathogenesis. From bacterial colonization to infection and through disease, the microenvironments of the host are in constant flux as bacterial and host factors contribute to changes at the host-pathogen interface, with the host attempting to eradicate bacteria and the bacteria fighting to maintain residency. A key component of this innate host response towards bacterial infection is the production of antimicrobial peptides (AMPs). As an early component of the host response, AMPs modulate bacterial load and prevent establishment of infection. Under quiescent conditions, some AMPs are constitutively expressed by the epithelium. Bacterial infection can subsequently induce production of other AMPs in an effort to maintain sterility, or to restrict colonization. As demonstrated in various studies, the absence of a single AMP can influence pathogenesis, highlighting the importance of AMP concentration in maintaining homeostasis. Yet, AMPs can increase bacterial virulence through the co-opting of the peptides or alteration of bacterial virulence gene expression. Further, bacterial factors used to subvert AMPs can modify host microenvironments and alter colonization of the residential flora that principally maintain homeostasis. Thus, the dynamic interplay between host defense peptides and bacterial factors produced to quell peptide activity play a critical role in the progression and outcome of disease.

## 1. Introduction

Antimicrobial peptides (AMPs) are widely distributed in animals and plants and are innate host defense peptides that display a broad spectrum of antimicrobial activity against bacteria, viruses, fungi and protozoa [1]. Lysozyme, lactoferrin, and collectins act primarily by targeting the bacterial outer membrane [2]. Other AMPs are cationic, amphipathic molecules of 12 to 50 amino acids in length that are capable of interacting with a bacterial cytoplasmic membrane, comprised primarily of negatively charged phospholipids. One of the ways in which these peptides can cause cell death is by inserting themselves into the cytoplasmic membrane to form channels that result in leakage of cytoplasmic contents [3,4,5]. In addition, multiple systems have been described that function to counter the initial lethality of rapid ion efflux (e.g., potassium) from the bacterial cell associated with AMP exposure [6,7]. Humans produce various types of AMPs, such as cathelicidins, thrombocidins and defensins [8,9,10,11,12].

In this review, we examine the impact of AMP production and dysregulation in select bacterial-mediated diseases and the consequences of AMP function in pathogenesis. Due to our research focus, we primarily focus the scope of this review to the discussion of the interplay of AMPs with bacterial pathogens. For a comprehensive look at bacterial strategies used to subvert AMP killing, Guilhelmelli and colleagues describe the current state of understanding pertaining to bactericidal mechanisms and bacterial responses to AMP exposure [13]. In addition, a number of reviews comprehensively characterize AMP expression within various anatomical locations of the human body, knowledge of which is important to consider to more fully understand the multifactorial nature and evolution of disease progression [14,15,16,17,18,19].

## 2. Modulation of AMPs during Viral Infection Facilitates Bacterial Disease

Many bacteria reside in the host as benign commensals, yet are able to relocate and colonize privileged, normally sterile sites of the body, particularly as a consequence of environmental stress. Translocation of commensal bacteria to a privileged niche occurs with the suppression of antimicrobial peptides (AMPs) that serve as one of the host’s first lines of defense. In the animal model for ascending otitis media (OM), the initial infection with respiratory syncytial virus suppresses expression of chinchilla β-defensin 1 (cBD-1), an ortholog of human β-defensin-3 (hBD-3), in the upper respiratory tract [20]. This suppression coincides with an increase in colonization of nontypeable *Haemophilus influenzae* (NTHI) in the chinchilla nasopharynx [21]. These data suggest that AMP production restricts levels of bacterial colonization within the normal flora population. Although a correlation between increased bacterial colonization with subsequent infection of the middle ear was not determined, the authors suggest this may explain the higher incidence of OM following viral infection and the polymicrobial nature of disease. In other work, rhinovirus increases the expression of elastase from neutrophils, and decreases expression of the secretory leukocyte peptidase inhibitor (SLPI) and elafin AMPs, enabling secondary *Staphylococcus aureus* colonization in humans with chronic obstructive pulmonary disease (COPD) [22]. Further, influenza type A suppresses TH17 effector cell pathway associated AMP production in mice exposed to *S. aureus*. The suppression of the TH17 pathway impairs bacterial clearance which leads to *S. aureus* colonization in the lung and subsequent development of pneumonia [23]. Viral infection is not, however, the only mediator of AMP dysregulation contributing to bacterial infection.

## 3. Bacterial Mechanisms of AMP Resistance

In a non-diseased state, pathogenic bacteria rely on AMP resistance mechanisms to survive the innate immune response and as such, initiate disease. Bacteria therefore have evolved a number of different strategies to sense and respond to various AMPs that they encounter at various sites in the body in order to survive.

### 3.1. Surface Charge Alterations

The most well characterized form of AMP resistance in Gram-negative bacteria is the modification of lipopolysaccharides (LPS) to decrease bacterial surface electronegativity. These changes reduce electrostatic attraction of positively charged AMPs, minimize bacterial association with AMPs, and decrease AMP-mediated killing. For example, the addition of phosphorylcholine (ChoP) to the oligosaccharide portion of NTHI lipooligosaccharide (LOS) results in increased resistance to the cathelicidin LL-37 [24]. Furthermore, the *dra* locus incorporates D-alanine to the outer membrane in *Bordetella pertussis* to similarly alter the surface charge and increase resistance to AMPs [25]. Highlighting the differential targets and killing ability of AMPs in NTHI, an *htrB* mutant, which switches from production of a hexaacylated lipid A to a tetraacyl lipid A, increases susceptibility of NTHI to hBD-2, but not to hBD-3 [26]. Finally, the deletion of *msbB*, which encodes a lipid A secondary acyltransferase in *Vibrio cholerae* El Tor strain C6706, increases susceptibility to polymyxin B, but interestingly does not affect *V. cholerae* classic strain O395 [27]. The difference in susceptibility to AMPs by closely related strains emphasizes the evolutionary differences that may enable bacteria to adapt to different environmental pressures.

Gram-positive bacterial cell walls lack LPS, but surface charge alterations still prove to be an important strategy for AMP resistance through modification of the lipoteichoic acids and cell wall teichoic acids that characterize their surface. Methicillin resistant *S. aureus* clinical isolates demonstrate an increase in resistance to LL-37 [28]. Additionally, the *dlt* operon, which is responsible for the D-alanylation of teichoic acids, increases resistance to a variety of AMPs. A *dlt* knockout mutant in *Enterococcus faecalis* is significantly more susceptible to the AMPs: nisin, polymyxin B, and colistin when compared to the wild type strain [29]. Furthermore, *dlt* knockout mutants in Group A *Streptococcus* (GAS) [30] and *S*. *aureus* [31,32] are more sensitive to AMP killing.

The importance of lipid decoration in the pathogenesis of Gram-negative bacterial-mediated disease is evident by studies in animal models of infection. A series of NTHI mutant strains that produce varying truncations of the sugar moiety of LOS are more susceptible to AMP killing and are attenuated when mice are infected via an intranasal route [33]. *Francisella* species with mutations in the genes that encode the deacetylase NaxD are unable to add galactosamine to lipid A and so are more susceptible to polymyxin B killing and are attenuated in a subcutaneous mouse model of infection [34]. A *Neisseria gonorrhoeae* mutant deficient in phosphoethanolamine decoration of lipid A is attenuated in the murine and human urogenital tract, likely due to an increase in susceptibility to AMPs [35,36]. Additionally, a *Salmonella* Typhimurium strain deficient in the incorporation of 4-aminoarabinose into lipid A, was less virulent when orally inoculated into mice as compared to mice infected with the wild type strain [37]. Further, *V. cholerae* *msbB* mutants showed a reduction in colonization rates compared to the wild type strain when inoculated intragastrically into infant mice [27]. Similar effects were observed in Gram-positive model systems. Survival of *Drosophila* *melanogaster* improves when infected with a *dltA* deficient strain of *S. aureus,* additionally, the *dltA* deletion mutant is less virulent in mice compared to the parental strain [38,39,40]. An increase in surface charge to reduce electrostatic AMP interactions is an essential and common mechanism for bacteria to limit AMP interactions, but this is only one of a variety of ways in which bacteria resist AMP killing.

### 3.2. Efflux Pumps

The active expulsion of AMPs from the bacterial cell via efflux pumps is utilized by many bacteria to avert lethality of AMP molecules and thus promote colonization of the host. An analysis of the *Vibrio parahaemolyticus* proteome indicated that TolC, a multiple drug resistance efflux pump, is significantly upregulated in strains resistant to AMPs, suggesting that this porin plays an important role in AMP resistance [41]. *Neisseria* species deficient in MtrCDE efflux pump proteins are more susceptible to killing by structurally diverse AMPs [42,43]. In addition, absence of the MefE/MefI efflux pump in *Streptococcus pneumoniae* increases susceptibility to LL-37 [44]. Likewise, in *Haemophilus ducreyi*, an *mtrC* mutant is more sensitive to both cathelicidin and β-defensin peptides [45]. Emphasizing the diversity of AMP resistance mechanisms between organisms, the *Escherichia coli* AcrAB and *Pseudomonas aeruginosa* MexAB efflux pumps, despite being similar to the *N. gonorrheoeae* MtrCDE efflux pump in substrates transported and amino acid identity, fail to play a significant role in AMP resistance [46]. Collectively, these data highlight the importance of efflux of AMPs in bacterial resistance to AMP lethality.

### 3.3. Import of AMPs into the Cytoplasm for Degradation

Recent investigations indicate that Haemophilus possesses a mechanism to actively import AMPs into the cytoplasm followed by intracellular degradation, thus serving to neutralize AMP-mediated lethality coupled with a nutritional benefit of amino acid recycling. The Sap (sensitivity to antimicrobial peptide) ABC transporter was identified through a transposon mutagenesis screen to identify *Salmonella typhimurium* strains that are more susceptible to the melittin and protamine AMPs [47]. In NTHI, *sapA* gene expression is up-regulated in the middle ear during NTHI-induced experimental OM [48]. A mutation in *sapA* significantly attenuates NTHI survival in the nasopharynx and the middle ear [49]. SapA binds the AMP cBD-1 [6] and mutants deficient in the Sap transporter permease proteins, SapB and SapC, lack the ability to transport AMPs to the bacterial cytoplasm, resulting in the accumulation of hBD-3 and LL-37 in the periplasm [50]. Thus, the Sap transporter binds and transports AMPs into the bacterial cytoplasm, for degradation, using proteases that have not yet been clearly elucidated. Importantly, neutralization of cBD-1 in the chinchilla middle ear restored virulence of the *sapA* mutant, indicating a critical role of this AMP resistance mechanism in *Haemophilus* pathogenesis [50]. Additional studies extended the importance of Sap-dependent import of AMP to *Haemophilus ducreyi*, the causative agent of canchroid in immunocompromised individuals. A mutant strain lacking the SapB and SapC permease proteins is more sensitive to LL-37 and is highly attenuated for pustule development in a human challenge model [51]. Interestingly, although SapA expression confers resistance to β-defensin and cathelicidin molecules in NTHI strains, *H. ducreyi* SapA does not appear to mediate defensin resistance. The observation of import of AMP molecules is now being investigated in other microorganisms. For example, in *E. coli,* the SbmA inner membrane transporter binds and imports the AMP Bac7 using an electrochemical transmembrane gradient [52]. It is intriguing to speculate that degradation of AMPs in the bacterial cytoplasm, subsequent to active transport, may provide a mechanism by which bacteria co-opt AMPs as a nutritional source to enhance their survival.

### 3.4. Secreted Proteins that Reduce AMP Activity

The mechanisms of bacterial resistance to AMP-mediated killing extend beyond surface modification and transport mechanisms, and include the active secretion of proteins that bind or degrade AMPs in an effort to abrogate their antimicrobial activity. Numerous studies indicate a role for exogenous proteases to neutralize the threat of host AMPs. Elastase production by *P. aeruginosa* was shown to be essential for evasion of LL-37 in chronic leg ulcer fluid [53]. Loss of metalloprotease SepA production in *Staphylococcus epidermidis* significantly impaired resistance to the dermcidin AMP [54]. In support of the importance of bacterial proteases in the development of disease, Puklo and colleagues observed degraded LL-37 in the gingival crevicular fluid that correlated with the presence of *Porphyromonas gingivalis*, *Tannerella forsythia*, and *Treponema denticola*, all of which express large amounts of proteases [55]. Furthermore, degradation of exogenous LL-37 also occurs in *P. gingivalis* infected gingival crevices [56]. Staphylococcal staphylokinase (SAK) and Streptoccocal streptokinase (SKA) are two exogenous proteins that activate the zymogen plasminogen into plasmin, which is essential for fibrinolysis. The use of these bacterial enzymes to dissolve blood clots has been proposed as a novel virulence mechanism for bacteria to evade confinement and to disseminate throughout the host. However, GAS can also use the plasmin to degrade LL-37 [57]. Similarly, in Staphylococcal induced pneumonia, cathelicidins are upregulated and the binding of SAK to host cathelicidins augmented fibrinolysis [58]. However, the role of AMPs in the augmentation of staphylokinase fibrinolysis is not an absolute. For example, α-defensin expression results in a significant decrease of fibrinolysis [59]. Collectively these data indicate that secretion of bacterial factors to neutralize host AMPs may provide an advantage for bacterial colonization at distinct environments in the host. It is also interesting to speculate that exogenous degradation of AMPs may provide a nutritional benefit to bacteria, as a source of amino acids for growth. Collectively, exogenous degradation of AMPs serves to limit bacterial surface interactions with AMPs, and so increase their survival.

### 3.5. Decoys That Sequester AMPs

The presence of bacterial factors such as LPS and DNA in the extracellular environment can serve as decoys to bind and neutralize AMPs and thereby reduce AMP mediated killing. Gram-negative bacteria produce a large amount of LPS and capsular polysaccharides, thus interaction of AMPs with these surface structures likely serve to delay bactericidal mechanisms. A study of major lung pathogens found that the polysaccharide components released by bacteria inhibited the activity of LL-37 and the cathelicidin-like protein SMAP-39; interestingly, the affinity of polysaccharides and AMPs is not based solely on electrostatic interaction as lower negative net charged particles do not correlate with better binding of LL-37 [60]. Furthermore, the presence of exogenous capsular polysaccharide from *Klebsiella pneumoniae*, *S. pnemuoniae*, and *P. aeruginosa* have demonstrated the capacity to serve as decoys by binding to AMPs and increasing bacterial resistance to polymyxin B and hNP [61]. Finally, plasmid DNA from *Shigella dysenteriae* reduces the expression of LL-37 and hBD-1 by epithelial cells, suggesting that early invading bacteria that are lysed, release factors that alter host defenses and enable subsequent invading bacteria to survive [62].

The bacterial biofilm matrix contains components that promote survival through associations with AMPs. Curli fibers, which are also known to be associated with *E. coli* biofilms, bind to LL-37, and thus prevent penetration, disruption, and killing of the bacterial biofilm [63]. *S. epidermidis* biofilms produce the extracellular polysaccharide intercellular adhesin, which increases bacterial resistance to a variety of AMPs including LL-37, hBD-3 and dermcidin [64]. The extracellular DNA component of *P. aeruginosa* and *S. Typhimurium* biofilm matrices chelate cations that normally repress the two-component PhoPQ and PmrAB signaling pathways and increases virulence [65,66]. When biofilm matrices are treated with DNase however, hBD-3 is more efficient at diminishing the biofilm formed by NTHI, again underscoring the importance of this matrix component in protection from AMP-mediated killing [67]. During biofilm growth, Gram-negative bacteria produce outer membrane vesicles (OMV) that contain proteins, phospholipids, and other periplasmic factors. The protein composition of OMVs released from *V. cholerae* is altered when strains are grown in the presence of sublethal concentrations of AMPs. One change is an increase in expression of the biofilm-associated extracellular matrix protein Bap1, shown to bind AMPs. A mutant deficient in Bap1 is more susceptible to AMP killing. These data suggest that the presence of Bap1 in OMVs is important for OMV associations with AMP that lead to increased AMP resistance [68]. Through diversionary tactics like LPS and DNA secretion, AMP bacterial interactions are lessened, allowing bacterial biofilms to persist in the host and further disease progression.

## 4. Colonization and Host Microenvironmental Factors That Influence AMP Activity

Transit of bacteria within the human host exposes the microorganism to various environments that vary in pH, nutrient concentrations, temperature, and host defenses. The environment of the host niche plays a pivotal role in the pathogenesis of disease, as a single new variable can drastically change the progression of disease in favor of the host or of the pathogen. For example, vitamin D has been linked to the host’s ability to remediate disease as TLR activation up-regulates expression of vitamin D hydroxylase and the vitamin D receptor, VDR. Activation of VDR through the vitamin D ligand leads to induction of expression of LL-37 [69]. VDR and LL-37 are likewise up-regulated in cirrhotic patients with spontaneous bacterial peritonitis, and cirrhotic patients with ascites exhibit vitamin D deficiency [70]. These data suggest that vitamin D could serve as an important factor in bacterial clearance. Conversely, some bacteria benefit from the effects of vitamin D for survival. For example, when vitamin D induces LL-37 production in human macrophages, GAS responds to subinhibitory concentrations of LL-37 through the CsrRS two-component system to increase expression of virulence factors (e.g., hyaluronic acid capsule, streptolysin O). These virulence factors then protect GAS from the killing effects of host cells [71]. Host body temperature also influences bacterial pathogenesis and AMP interactions. For example, as *Yersinia enterocolitica* moves from a lower temperature (representative of the environmental niche) to an elevated temperature (representative of the host niche), expression of the two-component systems PhoPQ and PmrAB decrease. This decrease in PhoPQ and PmrAB activity leads to a reduction in lipid A modification which increases bacterial susceptibility to AMPs [72]. Moreover, *Salmonella* species respond to increased pH through PhoPQ and PmrAB mediated expression of virulence genes that increase bacterial resistance to AMPs and facilitate transit through the gastric mucosa [73,74]. Conversely, expression of *phoQ* in *P. aeruginosa* is not activated by AMPs but is weakly activated by acidic environments. Therefore, *Salmonella* and *P. aeruginosa* have evolved different cues for PhoQ to sense the environment. PhoQ from *P. aeruginosa* is optimized to respond to the external microenvironment of the soil, which is both less acidic with less AMP exposure than that encountered by *Salmonella* [74]. Similarly, Enteropathogenic *E.* *coli* (EPEC) and Enterohemorrhagic *E.* *coli* (EHEC) have evolved differences in both the expression and function of the OmpT protease, shown to mediate AMP degradation in *E. coli*, due to the niche of each strain in the small and large intestines, respectively [75]. Thus, the human host is comprised of diverse microenvironments, each presenting a unique set of opportunities and challenges for pathogens in their continued effort to subvert host AMPs and maintain infection.

### 4.1. AMP Activity of the Skin

The skin serves as a protective barrier while maintaining a diverse micoflora. However, some bacteria can take advantage of breaches in this barrier to establish infection within the host. To prevent disease, it is important for the skin to maintain a healthy flora, in part, through the action of AMPs. While skin flora maintains homeostasis within the respective niches, foreign invaders can initiate changes that alter the microenvironment in favor of pathogen colonization. For example, *Finegoldia magna*, a skin commensal, maintains colonization through the action of the secreted protease SufA, which reduces the antibacterial activity of the MK and BRAK/CXCL14 AMPs. In contrast, the pathogen *Streptococcus pyogenes* protease SpeB demonstrates more efficient degradation of a wide range of AMPs, and thus favors colonization at this site [76]. The expression of neutrophil derived AMPs can likewise compromise the survival of commensals. LPS types 1A and 1B, from *Propionibacterium acnes*, stimulates hBD-2 expression in sebum producing skin epithelial cells or sebocytes. Whereas hBD-2 expression does not impact viability of *P. acnes*, the immunomodulatory effects of hBD-2 expression results in recruitment of neutrophils and induction of inflammation at the site of infection and can have unintended consequences for commensal survival [77]. It is increasingly clear that changes in the skin microenvironment that potentiate loss of benign commensals may lead to the replacement with pathogenic strains. Therefore, the homeostasis of commensals, governed by AMPs, is critical for the control of pathogenesis.

Lesions compromise the skin’s protective barrier and leave the host vulnerable to bacterial infection. When this protective barrier has been compromised, the role of AMPs in disease pathogenesis becomes critical. Mice deficient in an ortholog of LL-37 (CRAMP) infected with β haemolytic GAS display larger skin lesions and prolonged bacterial persistence compared to wild type mice. Furthermore, cathelicidin-resistant GAS is more persistent than cathelicidin-sensitive GAS in a post subcutaneous infection of mice [78]. The interplay between bacteria and AMPs impacts the microenvironment with critical consequences for the pathogenesis of skin disease. In acne vulgaris lesions, *P. acnes* secretes proteases that activate specific receptors that increase hBD-2 and LL-37 production, as well as a wide array of pro-inflammatory cytokines, contributing to epithelial lesions characteristic of this disease [79]. Likewise, skin lesions from acne inversa contain increased levels of hBD-2 in comparison to uninfected epithelial cells, further suggesting a role for hBD-2 in inflammatory skin disease pathogenesis [80]. Microbe-associated molecular patterns such as flagellin, stimulate host cell receptors that subsequently signal release of AMPs into the environment. Although *P.* *aeruginosa* flagellin stimulates the release of hBD-2 from keratinocytes, rhamnolipids secreted by *P. aeruginosa* inhibit release, effectively protecting the bacteria from hBD-2 mediated killing [81]. The role of AMPs in protecting the skin from pathogenic bacteria is most critical, as a breach in this line of defense can introduce bacteria into a multitude of new environments within the host.

### 4.2. Bacterial Homeostasis of the Nasopharynx

The contribution of AMPs in bacterial population management within the nasopharynx is crucial for human health, as carriage of commensal bacteria can influence the incidences of lower and upper respiratory disease. Lysozyme and hBD-1 and 2 contribute to the first line of defense by reducing the viability of major respiratory pathogens [82]. In addition, a reduction in the copy number of the DEFB-CN gene cluster, which encodes multiple beta defensins, leads to an increased risk for concurrent nasopharyngeal colonization with the OM pathogens NTHI, *Moraxella catarrhalis* and *S. pneumoniae* [83]. In support of the idea that AMPs influence residential bacteria critical for disease, the neutralization of cBD-1 increases carriage of NTHI in the nasopharynx [21]. This observation supports the idea that AMPs contribute to maintaining low levels of opportunistic pathogens, to protect from bacterial overgrowth and migration, which can lead to disease [21]. AMP exposure may also drive selection for strains that have a higher propensity to survive in this niche. For example, the presence of either LL-37 or hBD-1 selects for different *S. pneumoniae* isolates *in vitro*, suggesting a role for AMPs in establishment of commensal residents of the nasopharynx [84]. Levels of AMP expression have also been suggested to directly impact disease. For instance, among individuals colonized with *S. aureus*, more LL-37 is recovered from healthy controls than from patients with chronic rhinosinusitis, suggesting that dysregulation of LL-37 expression in the nasopharynx contributes to *S. aureus-*mediated disease [85]. Furthermore, carriers of *S. aureus* demonstrate increased expression of hNP-1, hNP-3, and hBD-2 in their nasal fluids, suggesting that the associations between *S. aureus* and these peptides are important in infection [86]. These data suggest that changes in the level of AMPs associated with inflammation due to invasive bacteria and the differences in AMP expression that exist between individuals impact the survival of normal commensals in the nasopharynx and so, may potentiate disease development.

### 4.3. AMPs and Lung Disease

The lungs have the second largest epithelial area exposed to the outside environment and the innate immune system is paramount to minimize bacterial invasion and infection. In the normal developing human lung, hBD-2 is the predominant defensin expressed. However, premature infants exhibit less expression of this critical AMP, which may contribute to increased susceptibility to infection [87]. β-defensins are important factors in host lung defense as demonstrated by the fact that hBD-2 and hBD-3 expression increases in human airway epithelial cells when stimulated by *S. pneumoniae*, an important causative agent of pneumonia [88]. The importance of cathelicidins in pathogenesis is shown by the upregulation of CRAMP in the murine lung following infection with *P. aeruginosa* and *K. pneumoniae*. In addition, CRAMP deficient mice infected with *K. pneumoniae* demonstrate reduced survival as well as an increase in bacterial persistence of *P. aeruginosa* and *K. pneumoniae* in the lungs [89]. Furthermore*,* bronchial epithelial cells stimulated with bacterial LPS demonstrate an increase in LL-37, which induces cellular apoptosis and IL-8 release, resulting in immune cell recruitment and a concomitant induction of an inflammatory response [90]. This proinflammatory response, mediated in part through AMP induction, likely contributes to chronic lung disease states such as COPD, pneumonia, and cystic fibrosis.

Microenvironmental alterations in AMP activity or concentration may also impact chronic lung diseases and facilitate residence by pathogenic bacteria which would further complicate disease progression. The environment of the cystic fibrosis lung is characterized as hyperinflammatory with a disrupted electrolyte composition due to a mutation in the chloride transporter CFTR*.* The potential for alterations in salt concentration during cystic fibrosis may impact the antimicrobial activity of AMPs. In fact, high salt concentrations have been shown to decrease the effectiveness of AMP killing [91,92]. High salt concentrations reduce activity of β-defensins [93,94], whereas LL-37 retains activity in high salt concentrations [95]. However, LL-37 activity is suppressed in the lungs of cystic fibrosis patients due to the interaction of LL-37 with bacterial-associated factors such as LPS, DNA as well as host glycosaminoglycans, and the dissociation of these complexes restores LL-37 potency [95,96]. The potential loss of LL-37 activity due to disease manifestation can leave the lung vulnerable to bacterial outgrowth or new bacterial invasion. Conversely, the presence of AMPs can also have unintended consequences for lung disease pathogenesis. For example, LL-37 induces IL-8, which in turn induces Muc5AC mucin production. Overproduction of Muc5AC can further compromise the airways in diseases such as COPD [97]. Exposure to LL-37 also selects for mutations in *P. aeruginosa* that facilitate mucoid conversion, making *P. aeruginosa* more recalcitrant to LL-37 killing [98]. Together these data underscore the importance of environmental factors, including AMPs, in the progression of lung disease.

### 4.4. AMPs and Periodontal Disease: Oral Cavity

The oral cavity serves as an entry point for pathogenic bacteria to enter the body; therefore, it is important for host defenses to prevent pathogenic bacteria from establishing residence within the oral cavity and causing disease. Periodontal disease is of particular concern, as this bacterial-mediated infection can lead to inflammation and the loss of both gum tissue and teeth. In an effort to eradicate pathogenic bacteria in the oral cavity, α-defensins hNP1-3 are significantly increased [55]. Although neutrophils are the primary source of hNP1-3 in infection, studies suggest that other sources are responsible for hNP1-3 secretion in later disease. For example, chronic periodontitis associates with significantly more hNP1-3 than aggressive periodontitis, yet both disease states shared similar neutrophil responses, which suggests an additional source of AMP secretion in chronic periodontitis [55]. This study highlights the fact that different stages of disease associate with expression of different AMPs that can have a direct influence on bacterial pathogenesis. The role of neutrophil-derived AMPs in maintaining health of the oral cavity is further suggested by the observation that patients with morbus Kostmann, a severe congenital neutropenia, are prone to chronic periodontal disease. When neutrophil production is stimulated in morbus Kostmann patients, the neutrophils do not express LL-37 and express lower concentrations of α-defensins [99]. In addition, dysregulation of β-defensin production also contributes to oral disease as a single nucleotide polymorphism in DEFB1, the gene that encodes hBD-1, has been linked to cases of periodontitis [100]. Furthermore, expression of hBD-2 mRNA increases in response to live *P. gingivalis* stimulation in a mouse human gingival graft model, which suggests a role for this AMP in prevention of periodontal disease [101]. Interestingly, hBD-3 plays a role in decreasing the deleterious effects of inflammation from disease, as hBD-3 is capable of binding and neutralizing the oral pathogen *P. gingivalis* via interaction with non-fimbral adhesin HagB, thereby interfering with binding of host cell receptors and thus decreasing inflammation [102]. Dysregulation of any of the multitude of AMPs implicated in oral diseases appears to play an important role in the progression of disease.

### 4.5. AMPs and the Gastric Mucosa

Invading bacteria are normally prevented from colonizing the gastric mucosa through the action of acidic gastric juice, mucus, and AMPs. Despite these harsh conditions, some bacteria are able to circumvent the antimicrobial conditions unique to this microenvironment by responding to environmental cues and changing expression of AMP resistance genes or by disrupting the host environment. *Helicobacter pylori* alters the expression of several key AMPs known to be associated with the gastric mucosa. hBD-2 expression increases in response to bacterial insult, suggesting a role for this peptide in controlling the pathogenesis of *H. pylori* mediated diseases [103,104,105]. There are conflicting reports in the literature regarding the regulation of transcription of hBD-1, hBD-3 and LL-37 in the gastric mucosa in response to bacterial insult. hBD-1 is constitutively expressed in the normal gut, but may be either upregulated by *H. pylori* infection [106], down regulated by *H. pylori* infection [105] or unchanged between infected and non-infected gastric mucosa [103]. Expression of hBD-3 and LL-37 may be increased [107,108], decreased [104] or undetectable in the gastric mucosa [103] following *H. pylori* infection. The different AMP expression levels observed is likely due to the use of different *H. pylori* strains and the presence of the virulence factor CagA, which can translocate into host cells and inhibit synthesis of hBD-3 via dephosphorylation of epidermal growth factor receptor [109]. Despite these contradictory findings, we can speculate that modulation of the bacterial effects of hBD-3 and LL-37 can regulate infection by *H.* *pylori* in the gastric mucosa and thus be a critical defense mechanism against *H. pylori* pathogenesis [103].

### 4.6. Homeostasis and Diseases of the Intestines

The intestines are home to a diverse micro-flora that evolves to withstand AMP killing, since AMPs are routinely expressed in the intestines. Paneth cells continually express AMPs, such as HD-5, HD-6 and lysozyme that serve as a first line of defense against invading bacteria [110]. Upon infection with *V. cholerae* [110] *Bacteroides fragilis* [111] and various *Pseudomonas* species [112], hBD-2 is upregulated, which suggests that this β-defensin is important in the modulation of disease. CRAMP deficient mice are more susceptible to *Citrobacter rodentium* colonization, epithelial damage and systemic infection, which further implicates a role for AMPs in the small intestine in preventing bacterial pathogens from causing dysbiosis [113]. The influence of AMPs within the intestinal environment has also been demonstrated in mice deficient in α-defensins, as loss of these AMPs leads to significant differences in the composition of intestinal microbiota, although the total bacterial load remains unaffected [114]. Similarly, mice deficient in α-defensins are more attenuated in removal of *Chlamydia trachomatis* from the small intestines [115]. Combined, these studies suggest a role for AMP influence on population shifts, which may impart susceptibility or resistance to diseases of the intestinal tract. In a mouse model of necrotizing enterocolitis, where AMP levels and intestinal inflammation increase, the probiotic *Bifidobacterium bifidum* attenuates disease and decreases AMP levels, by normalizing the microbiota and re-establishing the synergistic relationship between the commensal and the host [116]. However, the influence of AMPs on normal commensal populations may not always be the result of direct AMP and bacterial interactions. For example, LL-37 stimulates mucus production in the human colonic cell line HT-29 and the administration of CRAMP upregulates expression of mucin genes in the colonic tissue of mice with experimentally induced colitis. These data demonstrate that LL-37 can influence the microenvironment of the intestines by stimulating the production of mucus [117,118]. As cathelicidin deficient mice have less thick and non-homogenous mucus layers when compared to wild type animals, *E. coli* are more likely to penetrate the mucus layer and attach to the underlying epithelial cells [119]. The intestinal mucus is therefore an important barrier that prevents bacteria from reaching the intestinal epithelium, so any deficiencies in the production could have a critical impact on bacterial pathogenesis in the intestines. Finally, the scarce availability of oxygen in the intestines is important to consider in AMP-mediated killing. For example, HD-5, is effective at killing facultative anaerobic species, but is ineffective at killing strict anaerobic bacteria, while hBD-3 is only able to kill anaerobic species under aerobic conditions [120]. Therefore, bacterial residents of the intestines display differential susceptibility to AMP killing dependent on their metabolism or O_2_ availability, further emphasizing the complex interactions between AMPs and bacteria in pathogenesis. The maintenance of balance between host factors, nutrient status, and commensal populations in the bowel is important to consider particularly with inflammatory bowel diseases such as Crohn’s disease and ulcerative colitis.

### 4.7. Urinary Tract Infections

The environment of the urinary tract is generally considered sterile, despite the close proximity to the gastrointestinal tract, and provides unique challenges for invading bacterial attachment due to the shedding of epithelial cells, urine flow, and expression of innate defenses such as AMPs. Cathelicidins are constitutively expressed by both human and mouse urinary epithelia. The importance of cathelicidins in preventing pathogenic bacteria from establishing disease is demonstrated by the observation that cathelicidin resistant *E. coli* are more likely to invade the urinary tract than cathelicidin susceptible strains, while CRAMP deficient mice are more easily infected by *E. coli* when compared to wild type mice [121]. Additionally, defensins in the urinary tract have diverse functions in the protection of the urothelium from disease. In patients undergoing urinary diversion surgery, there is a significant increase in the expression of HD-5 α-defensin and decrease in expression of hBD-1 in urothelial tissue [122]. As HD-5 shows bactericidal activity towards uropathogenic *E. coli* (UPEC), these data suggest that this peptide mediates a direct role in bacterial clearance in urinary tract infection (UTI) pathogenesis. Becknell and colleagues have observed a reduction in mBD-1 expression in the mouse bladder in response to UPEC infection and furthermore, observed that a mouse deficient in mBD-1 has no significant impairment when challenged with UPEC, suggesting that mBD-1 may not contribute to the control of UPEC mediated infection [123]. In contrast, mBD-3 and mBD-14 display dose-dependent bactericidal activity to UPEC *in vitro*, suggesting a role for AMPs in mucosal immunity in the lower urinary tract [123]. RNase6 and 7 are members of the RNaseA superfamily, and have recently been described as having important roles in urinary tract defense where they exhibit strong antimicrobial activity towards uropathogenic bacteria. RNase7 is important in the urinary tract due to the observation of an increase in levels in urine samples obtained from patients with pyelonephritis as compared to healthy controls [124]. While RNase7 is significantly increased in relation to RNase6 in non-infected human bladder and kidney tissues, RNase6 expression significantly increases during pyelonephritis due to the influx of immune cells [125]. The urinary tract produces multiple classes of AMPs that function in homeostasis of the sterile urinary tract as well as being responsive to disease.

## 5. Host-Pathogen Tug of War

After a bacterium colonizes a privileged location, the development and subsequent progression of disease is dependent on the successful management of increasingly complex interactions between the host and the bacterium. Convention originally stated that positively charged AMPs primarily exert activity by interaction with negatively charged bacterial surfaces leading to bacterial cell lysis. However, it is becoming increasingly apparent that AMP function is multifactorial. For example, bacteria respond to the presence of AMPs through changes in bacterial gene expression as a result of signaling events. Exposure of *E. coli* to sublethal concentrations of cecropin A changes the transcriptional profile of a subset of genes [126]. Certain AMPs are capable of mediating changes in gene expression profiles of bacterial virulence factors and as such, may influence the dynamics of the pathogen-host interaction. In addition, bacterial responses to environmental pressures may affect host responses and ultimately, may impact disease remediation or continued bacterial infection.

### 5.1. AMPs Influence Bacterial Gene Expression 

Although the host responses and subsequent microenvironmental changes are intended to eradicate infection, pathogenic bacteria sense and respond to environmental cues to regulate transcription of virulence factors. One such environmental cue is the secretion of AMPs by the host. Importantly for the bacterium, transcription of gene products that contribute to mechanisms of AMP resistance are often upregulated upon bacterial exposure to AMP molecules. A classic example is the PhoPQ regulatory system, a well-studied two-component system that responds to environmental stressors and modulates expression of genes that contribute to modification of LPS, leading to increases in AMP resistance in Gram-negative bacteria [127,128]. Although best described in *S. typhimurium* [129], a role for PhoPQ has been shown for AMP resistance in *P. aeruginosa* [130], *E. coli* [131], *K. pneumoniae* [132], *Shigella flexneri* [133], *Neisseria meningitidis* [134] and *Yersinia pestis* [135]. The conservation of gene function across different species highlights the importance of this mechanism for AMP resistance. Furthermore, PhoPQ regulates expression of another two-component system, PmrAB. PmrAB is involved in the regulation of expression of *pmrE* and *pmrHFIJKLM*, the products of which modify lipid A with 4-aminoarabinose [37,136]. A *S.* Typhimurium *pmrF* insertional mutant, lacking the 4-aminoarabinose modification, displays a decrease in virulence in the mouse when administered orally [37]. While *S.* Typhimurium requires the PmrAB two-component system for regulation of *pmrF* expression, regulation of *pmrF* in *Yersinia pseudotuberculosis* occurs solely through the PhoPQ pathway. Furthermore, *pmrF* expression is not essential for either AMP resistance or survival of *Y. pseudotuberculosis* in the mouse [137]. While the PhoPQ and PmrAB systems are perhaps the most well studied two-component systems involved in AMP resistance in Gram-negative bacteria, other two-component regulatory systems such as ParRS, CprRS, and ColRS increase resistance of *P. aeruginosa* to AMPs through alterations in surface charge [7,138,139].

Gram-positive bacteria also use two-component systems to respond to the presence of AMPs as a way to regulate resistance to bactericidal activity. For example, in *S. aureus* attack by certain AMPs, such as HNP-1 cause a disruption in membrane potential that activates the LytSR two-component system which is protective against AMPs *in vitro* [140]. In addition to the LytSR system, AMPs induce the GraSR regulon (also known as the AMP sensor, or ApsSR) of *S. aureus*, which up-regulates expression of the virulence genes *dlt*, *mprF*, and *vraFG*, and so increases resistance to AMPs [141,142]. Similarly, in GAS, the well-studied two-component system CsrRS (also known as CovRS) can sense LL-37 and alter the expression of virulence genes and thereby increase the resistance to phagocytic killing of this bacterium [143]. Additionally, during progression of disease, GAS transitions to a mucoid phenotype though upregulation of hyaluronic acid capsular polysaccharide biosynthetic enzymes via CsrRS sensing of LL-37 [144]. Finally, growth of *S. pyogenes* in the presence of LL-37 is dependent on the CovRS two-component system *in vitro*. CovS inactivates CovR repression of the *dlt* operon. The *dlt* operon encodes proteins responsible for D-alanylation of the anionic polymers of lipoteichoic and wall lipoteichoic acids. Increased expression of *dlt* may ultimately produce a cell wall with a higher positive charge density and prevent binding of cationic AMPs [145]. The sensing of AMPs and increases in expression of AMP resistance mechanisms via two-component systems allow bacteria to adapt to the innate immune response and persist within the host while establishing infection.

Although regulation of two-component signaling pathways confers AMP resistance to many bacteria, there are additional mechanisms that increase bacterial resistance to AMP molecules. For example, exposure of *P. aeruginosa* to sublethal concentrations of LL-37 increases expression of a number of virulence factors. These virulence factors include genes whose products encode multidrug efflux pumps and those involved in LPS modification as well as in the production of quorum sensing molecules [146]. Host production of the anionic AMP dermcidin increases the expression of the global regulator Agr and decreases the expression of the global regulator SarA in *S. epidermidis*. Regulation of gene expression by Agr and SarA subsequently leads to enhancement of dermcidin proteolysis and thus is protective toward *S. epidermidis* [54]. AMP exposure increases expression of transporters that actively export AMPs to remove them from the cell or, alternatively, import AMPs for degradation and so confer resistance [44,48]. In *P. aeruginosa*, sublethal concentrations of polymyxin B upregulates the expression of *psrA*, which increases AMP resistance through regulation of swarming, motility and biofilm formation [147]. Finally, *in vitro* exposure of *P. aeruginosa* to LL-37 selects for a mutation in the negative regulator of mucoidy, MucA, and so increases alginate synthesis. This increase in alginate production converts bacteria to a mucoid phenotype that provides protection against host AMPs. Moreover, this LL-37 induced mutation in MucA parallels what is observed in the cystic fibrosis lung [98]. Collectively, these examples illustrate how bacterial sensing of AMPs can lead to recalcitrance and so may prove to be the tipping point in the progression of disease.

### 5.2. AMPs Influence Disease Progression

Whereas regulation of transcription controls microbial virulence mechanisms in response to AMP exposure, the consequence of these responses on pathogenesis continues to be explored in relevant animal models of disease (see Table 1). For example, in the lumen of the mouse intestine, *S.* Typhimurium uses the PhoPQ and PmrAB two-component systems to respond to insult by CRAMP, which indicates the importance of PhoPQ and PmrAB in the pathogenesis of *S.* Typhimurium [148]. In further support of this idea, inactivation of the PhoPQ system of *S. typhimurium* leads to a decrease in survival in the *Caenorhabditis elegans* intestine. The loss of survival is abrogated when expression of the *C. elegans* AMP Spp-1 is reduced with RNAi treatment, suggesting that PhoPQ dependent resistance to AMPs is vital for survival in the host [149]. GAS mutants deficient in the transcriptional regulator RALP3 are more sensitive to mCRAMP and attenuated in a murine model of infection [150]. *S. aureus* strains lacking the AMP sensing protein ApsS (also known as GraS) are significantly attenuated for infection of the kidneys when intraperitoneally injected into the mouse [141]. Finally, *S. aureus* strains deficient in LytS of the LytSR two-component system are more susceptible to exogenous AMPs in a rabbit model of aortic infective endocarditis [140]. Together, these data suggest that bacterial responses to host defense are critical for the ability to withstand the host immune factors generated as a consequence of disease.

The interactions between bacteria and AMPs are complicated by the introduction of additional host factors as disease persists. AMPs induce immunomodulatory changes in the host in an effort to resist bacterial colonization. The stimulation of epithelial cells by LL-37 increases production of the chemokine IL-8, which leads to the recruitment of immune cells. This influx of immune cells leads to inflammation and the induction of further changes in the microenvironment. In the case of COPD, these inflammatory changes can become persistent and irreversible and thereby influence pathogenesis [90]. AMPs also provide a key function in immune cell recruitment as hBD2-4 and LL-37 induce human keratinocytes to produce the pro-inflammatory cytokine IL-18 [151]. In addition to pro-inflammatory cytokines, a diverse array of AMPs are produced as the disease progresses through bacterial stimulation of AMP release from epithelial cells and α-defensin release from the recruited immune cells. In healthy individuals, α-defensins are introduced into the environment primarily by neutrophils, but an alternative mechanism may be responsible for the increased levels of α-defensins in late stages of chronic disease [55]. Additionally, in both the non-infected human bladder and kidney, RNase7 provides one of the first lines of defense against pathogens; however, expression of RNase6 significantly increases upon bacterial insult [152]. Furthermore, expression of RNase7 and hBD-2 increases early during oral bacterial biofilm formation on human gingival epithelial cells, but expression declines as biofilms mature [153]. AMPs that may prove to be important in the acute stages of infection may be less important in chronic infections, possibly due to bacterial adaptation to the environment. For example, as bacterial biofilms mature they release extracellular DNA (eDNA) that associate with AMPs and confer AMP resistance. AMPs that are effective at bacterial clearance during the early stages of disease, when the levels of eDNA are too low to have an impact, may lose effectiveness once the biofilm matures, primarily due to neutralization of AMP through eDNA association [67]. The chronology of AMP production during disease and consequence upon bacterial interaction in a rapidly changing inflammatory environment can significantly influence disease progression in the host. Understanding a role for AMP activity during acute and chronic disease states continues to be a significant challenge, but one that remains of importance to understand a therapeutic value for AMP interventions in disease.

**Table 1:** *In vivo* Models of antimicrobial peptides (AMPs) in Disease.

Disease Site	Animal Model	Host AMP Neutralized	Bacteria	Gene	Source
Disseminated	*Drosophila*, Mouse	---	*S. aureus*	*dltA*	[39,40]
	Mouse	---	*Francisella tularensis*	*naxD*	[34]
	Mouse	---	*S.* Typhimurium	*pmrF*	[37]
	Mouse	---	*Y. pseudotuberculosis*	*pmrF*	[137]
	Mouse	---	GAS	*Ralp3, lsa*	[150]
	Rabbit	---	*S. aureus*	*lytS*	[140]
Intestine	Mouse	CRAMP	*E. coli*	*---*	[119]
		---	*S. aureus*	*dltA*	[38]
		α-defensins	*---*	---	[114]
		CRAMP	*C. rodentium*	*---*	[113]
		α-defensins	*C. trachomatis*	---	[115]
		CRAMP, α-defensins	*S.* Typhimurium	*phoPQ* and *pmrAB*	[148]
		---	*V. cholerae*	*msbB*	[27]
Kidney	Mouse	---	*S. aureus*	*apsS*	[141]
Lung	Mouse	CRAMP	*K. pneumoniae*, *P. aeruginosa*	*---*	[89]
Middle Ear	Chinchilla	CβD-1	NTHI	*sapA*	[50]
Nasopharynx	Chinchilla	CβD-1	NTHI	*---*	[21]
Skin	Human	---	NTHI	*sapBC*	[51]
	Mouse	CRAMP	GAS	*---*	[78]
Stomach	*C. elegans*	Spp1	*S. typhimurium*	*phoPQ*	[149]
Urogenital Tract	Mouse, Human	---	*N. gonorrhoeae*	*lptA*	[35,36]
	Mouse	CRAMP	*E. coli*	*---*	[121]

### 5.3. Using What We Know About AMPs for Potential Therapeutic Use

It is clear that AMP molecules play a significant role in host–pathogen interactions that influence the establishment and course of disease progression. In addition to the bactericidal properties of AMPs, recent investigations support the use of AMPs as inflammatory modulators to reduce the pathological consequences associated with disease. For example, AMPs bind bacterial surface molecules to prevent interactions with host cell receptors and thus attenuate pro-inflammatory cytokine responses. LL-37 binds *N. meningitidis* capsular polysaccharides and blocks TLR2 and TLR4-MD2 dependent activation of pro-inflammatory cytokine release in human and murine macrophages [154]. In other studies, treatment of mice with CRAMP reduces intestinal inflammation in response to *Clostridium difficile* toxin A [155]. In addition, hBD-3 and LL-37 reduce IL-8 secretion from gingival fibroblast cells exposed to *Aggregatibacter* *actinomycetemcomitans* lipopolysaccharide [156]. The suppression of a pro-inflammatory cytokine response, through delivery of AMPs, also correlates with bacterial clearance and disease severity. In a mouse model of methicillin-resistant *S. aureus* (MRSA) induced pneumonia, LL-37 neutralization of lipoteichoic acid induces pro-inflammatory cytokine release resulting in clearance of bacterial infection [157,158]. Similarly, temporin B and royal jellein 1, secreted from the European red frog and honeybees, respectively, act synergistically to reduce inflammation through down regulation of the pro-inflammatory cytokines TNF-α and IFN-γ and upregulation of the anti-inflammatory cytokine IL-10. [159]. Topical application of the king cobra derived AMP OH-CATH30 significantly decreases release of the pro-inflammatory cytokines TNF-α and IL-1β coinciding with a marked reduction in corneal epithelial loss [160].

Whereas AMPs attenuate inflammation associated with disease, as described above, evidence also indicates that AMPs stimulate inflammatory responses in an effort to eradicate bacteria from the host. In a murine model of *P. aeruginosa* lung infection, exogenous LL-37 increases recruitment of neutrophils to the lung, which results in bacterial clearance [161]. Defensins and LL-37 can also induce secretion of the pro-inflammatory cytokine IL-31, as well as other pro-inflammatory cytokines involved in the innate immune response from human mast cells [162]. These immunomodulatory activities of AMP molecules appear to differ based upon the concentration of peptide. When human epithelial cells are stimulated with LPS, low concentrations of LL-37 suppress IL-8 secretion while greater concentrations of LL-37 induce IL-8 secretion [163]. Furthermore, high concentrations of LL-37 either augment or antagonize inflammatory cytokine responses depending on the presence of specific immune mediators [164]. The immunomodulatory properties of AMPs highlight their multifunctional roles in the pathogenesis of disease independent of bacterial killing and may prove to be useful for potential therapeutic application.

In addition to the use of naturally derived AMPs or modification of existing AMPs, synthesis and utilization of entirely new AMP structures shows promise in treating bacterial infections. For instance, a truncated variant of LL-37 lacking the six C-terminal residues is more effective at killing planktonic and biofilm-associated *Burkholderia pseudomallei* than LL-37 or LL-37 derived fragments [165]. Furthermore, P60.4Ac and P10, synthetic derivatives of LL-37, are more efficient than LL-37 in the eradication of MRSA in a human skin model of infection [166]. Similarly, the synthetic peptides E2, E6, and CP26 are more effective than CRAMP and LL-37 at killing *Mycobacterium tuberculosis* and *P. aeruginosa in vitro* and importantly, are more efficacious in the control of *M. tuberculosis* in the mouse lung, which supports a therapeutic use for these peptides [167]. Synthetic AMPs may also demonstrate the capacity to act in an immunomodulatory manner similar to naturally produced AMPs. For example, the synthetically derived A3-APO decreases *S. aureus* load in a murine burn wound model and decreases the burden of *P. acnes* in an intradermal mouse ear model despite not directly killing the strains *in vitro*, suggesting that immunomodulation by A3-APO of the host microenvironment contributed to remediation of disease [168]. The efficacy of AMPs in disease treatment can be further improved through peptide modification. For example, the covalent linking of polyethylene glycol through the process of PEGylation, reduces the interactions of AMP molecules with both host cells and host enzymes, and so focus AMP interaction with the bacterial cell surface. In addition, PEGylation decreases the cytotoxicity of modified AMPs toward host cells. The PEGylation of the synthetic AMP CaLL, designed to incorporate regions from cecropin A and LL-37, decreases AMP elicited cytotoxicity of epithelial cells in an *ex vivo* perfused lung model [169]. When considering designing novel therapeutics based on LL-37, future studies should be cognizant that inflammatory environments contain the enzymes PAD2 and PAD4, which cause the deamination (citrullination) of LL-37 arginine residues. These residue changes impair the ability of LL-37 to bind LPS and fail to prevent septic shock in D-galactosamine-sensitized mice injected with LPS. A modification to prevent residue citrullination could potentially increase LL-37 efficacy in inflammatory environments [170]. Modification or synthesis of AMPs that select for improved killing and reduced host cell damage may prove to be a beneficial therapeutic tool for a variety of bacterial diseases where other therapies have failed.

The combination of AMPs with antibiotics or vitamins can enhance bactericidal activity. The oral administration of vitamin 25D3, a vitamin D precursor, increases cathelicidin production in bladder cells exposed to UPEC [171]. In addition to vitamin D, parathyroid hormone (PTH) can act in a synergistic manner to increase cathelicidin expression. The addition of PTH to mouse skin reduces susceptibility to GAS infection. Further, this effect was diminished in mice on a vitamin D restricted diet, suggesting an important role for the combination of PTH and vitamin D in pathogenesis [172].

Susceptibility of bacteria to AMP molecules varies widely based on both the bacterial species and the microenvironment of the disease. As already discussed, in the cystic fibrosis lung, high salt concentrations can diminish activity of some AMPs. SMAP29 and CAP18, however, are not susceptible to high salt concentrations and therefore are most effective at killing *P. aeruginosa*, *E. coli* and *S. aureus* strains. These AMPs may thus be attractive therapeutics for the remediation of bacteria in cystic fibrosis patients [173].

As antibiotic resistance increases, novel therapies are needed to treat bacterial infections. The combinatorial use of AMPs and antibiotics has proven effective. When combined with antibiotics, hBD-3 and LL-37 act in a synergistic manner to kill toxinogenic and non-toxinogenic *C. difficile* strains *in vitro* [174]. Similarly, α-helical AMPs in combination with antibiotics demonstrate additive killing against Gram-negative and Gram-positive bacteria *in vitro* [175]. Finally, the combination of the synthetic AMP GL13K and tobramycin prove more effective in eradication of *in vitro* grown *P. aeruginosa* biofilms than either GL13K or tobramycin alone [176]. The AMP PL-5 likewise synergized with levofloxacin hydrochloride in a mouse model of *S. aureus* infection [175]. The use of combinatorial therapies to treat biofilm-related diseases has proven effective. Colistin targets metabolically inactive *P. aeruginosa* located deep within the biofilm; whereas the antibiotics ciprofloxacin and tetracycline target the outermost subpopulation of the *P. aeruginosa* biofilm that are more metabolically active [177]. As bactericidal mechanisms differ between AMP and antibiotics, the combinatorial use will likely decrease the emergence of antibiotic resistant strains. The continued study of AMP interactions in the host throughout pathogenesis is vital for continued design of implementation strategies or novel AMP structures, which may provide treatment for burdensome bacterial diseases that present recalcitrance to traditional therapies.

## 6. Conclusions

**Figure 1:**
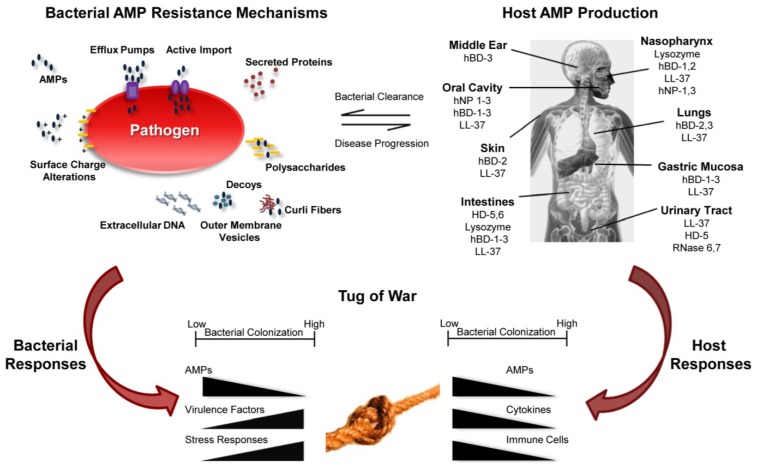
Summary of host-pathogen interaction as a consequence of AMP function. Bacterial AMP resistance mechanisms provide advantages to pathogens (upper left) leading to disease progression. The host produces AMPs to control bacterial growth leading to bacterial clearance (upper right). AMPs modulate gene expression by both host and pathogen resulting in a tug of war to shift the balance between health and disease.

As bacteria navigate through the human host they are confronted with host AMPs that threaten their survival (Figure 1). By reliance on suppression of AMP activity or mediation of AMP resistance factors, bacteria can persist to establish residency in a new microenvironment. The colonization of a foreign invader disrupts environmental homeostasis which results in the increased expression of host defense factors, including AMPs. As bacteria sense the presence of AMPs, virulence gene expression is increased to potentiate survival. These changes in gene expression increase bacterial persistence and can modulate disease severity. As bacterial residence is maintained, the environment continues to evolve as new AMPs are produced in response to a changing inflammatory response. Inflammation, nutrient status, and the host microflora influence the interplay between pathogenic bacteria and host AMPs. This complex network of interdependent interactions is in constant flux with slight changes in variables dramatically impacting the course of disease. Here, we have summarized a sampling of the complex interactions between bacteria and host throughout pathogenesis. A better understanding of mechanisms of host defense, production of environmental cues during infection, and the evolving host response to maintain bacterial homeostasis in the face of foreign invaders intent on dysbiosis, will direct design of therapies to alleviate disease.
